# PERK/*EIF2AK3* integrates endoplasmic reticulum stress-induced apoptosis, oxidative stress and autophagy responses in immortalised retinal pigment epithelial cells

**DOI:** 10.1038/s41598-022-16909-6

**Published:** 2022-08-03

**Authors:** Neil Saptarshi, Louise F. Porter, Luminita Paraoan

**Affiliations:** 1 Department of Eye and Vision Science, Institute of Life Course and Medical Sciences, University of Liverpool, Liverpool, UK; 2 Laboratoire de Génétique Médicale, Institut de Génétique Médicale d’Alsace, INSERM U1112, Fédération de Médecine Translationnelle de Strasbourg (FMTS), Université de Strasbourg, Strasbourg, France; 3 Service de Génétique Médicale, Institut de Génétique Médicale d’Alsace, Centre de Référence pour les Affections Rares en Génétique Ophtalmologique (CARGO), Strasbourg, France; 4 Present Address: Department of Biology, Faculty of Arts and Sciences, Edge Hill University, Ormskirk, UK

**Keywords:** Cell biology, Molecular biology

## Abstract

Retinal pigment epithelium (RPE) performs essential functions for ensuring retinal homeostasis and is a key site for pathogenic changes leading to age-related macular degeneration (AMD). Compromised proteostasis in RPE results in ER stress and ER stress-dependent antioxidant, apoptosis and autophagic responses. ER stress induces the unfolded protein response (UPR) in which *EIF2AK3,* encoding the protein kinase RNA-like ER kinase (PERK), acts as a key regulator. Downregulated *EIF2AK3* gene expression has recently been identified in AMD using human donor RPE, however the molecular mechanisms that integrate the various ER-mediated cellular pathways underpinning progressive RPE dysfunction in AMD have not been fully characterised. This study investigated the downstream effects of PERK downregulation in response to Brefeldin A (BFA)-induced ER stress in ARPE-19 cells. PERK downregulation resulted in increased ER stress and impaired apoptosis induction, antioxidant responses and autophagic flux. ARPE-19 cells were unable to efficiently induce autophagy following PERK downregulation and PERK presented a role in regulating the rate of autophagy induction. The findings support PERK downregulation as an integrative event facilitating dysregulation of RPE processes critical to cell survival known to contribute to AMD development and highlight PERK as a potential future therapeutic target for AMD.

## Introduction

The retinal pigment epithelium (RPE) is a monolayer of post-mitotic epithelial cells that forms the outermost aspect of the retina^1^. Its critical role in maintaining retinal homeostasis is ensured by key and specific functions involving endoplasmic reticulum (ER)-mediated proteostasis^2^. These include regulation of metabolites and nutrient directional transport between the basally located choroid, the outer retinal blood supply and the apically located photoreceptors, modulation of light- and intense metabolism-induced oxidative stress, daily phagocytosis of photoreceptor outer segments, and maintenance of the outer blood-retinal barrier^1,3^. In addition, the apical and basal cellular environments formed by the RPE are maintained through tight regulation of the intracellular trafficking and polarised secretion of proteins^2^.

RPE dysfunction is a key factor contributing to the development of age-related macular degeneration (AMD), the foremost cause of vision loss in adults in developed countries^1,4–6^. While the greatest risk factor for AMD is ageing, various environmental risk factors (cigarette smoke, light exposure) have been linked to dysregulated ER stress-dependent antioxidant responses, apoptosis and autophagy^7–11^. Furthermore, various proteins and protein variants associated with increased risk for developing AMD are likely contributors to ER stress and the ensuing compromised proteostasis in the RPE due to impaired intracellular trafficking, abnormal processing/accumulation/aggregation and/or reduced RPE secretion^12–15^. However, the actual molecular determinants that integrate the various ER-mediated cellular pathways underpinning progressive RPE dysfunction, which contribute to the AMD pathogenesis have not been fully characterised.

We and others have recently identified significant dysregulation of *EIF2AK3* gene expression in the RPE of human donors with early and intermediate AMD^16,17^. *EIF2AK3* encodes the protein kinase RNA-like ER kinase (PERK), a key regulator of the unfolded protein response (UPR) in response to ER stress^8,9^. In addition, significant enrichment of antisense RNA transcripts of the PERK-EIF2 has been shown in AMD RPE, suggesting that downregulated *EIF2AK3* gene expression and PERK pathway signalling play a role in the pathogenesis of early/intermediate AMD in the RPE^16,17^.

The ER plays a key role maintaining proteostasis, responsible for the biosynthesis and post-translational modifications of secretory and membrane proteins^10^. Accumulation of misfolded proteins and a failure of the classical secretory pathway induces ER stress and the UPR^18^. The UPR is an adaptive response aimed at restoring ER homeostasis, governed by three ER-membrane localised proteins: activating transcription factor 6 (ATF6), inositol-requiring enzyme 1 (IRE1) and PERK^19^. Glucose-regulated protein 78 (GRP78) functions as a key sensor of ER stress^19^. Upon sensing misfolded proteins, GRP78 disassociates from the *N*-terminal domains of ATF6, IRE1 and PERK resulting in the induction of UPR signalling cascades^19^. In the canonical PERK pathway of the UPR, GRP78 disassociation induces PERK auto-phosphorylation^19^. Phosphorylated PERK subsequently phosphorylates eukaryotic translation initiation factor 2 alpha (eif2α), to attenuate global protein translation and reduce incoming ER protein load via upregulated ER chaperone expression^19^. Furthermore, PERK has been shown to phosphorylate nuclear respiratory factor 2 (Nrf2), which upregulates antioxidant genes such as NAD(P)H Quinone Dehydrogenase 1 (NQO1) in response to ER stress^9,20^. Failure of the adaptive UPR initiates selective PERK-dependent upregulation of the pro-apoptotic transcription factor C/EBP homologous protein (CHOP)^19^. CHOP upregulation is associated with downregulation of the pro-survival B-cell lymphoma 2 (Bcl2) protein and upregulation of pro-apoptotic Bcl2-associated protein X (Bax) and caspase cascade activation^21–23^. PERK is also crucial in regulating the induction of autophagy and previous investigations have described impaired autophagy induction in response to serum starvation in AMD RPE^23–25^. Of note, treatment options based on facilitating increased PERK expression suggested to alleviate causal factors in neurodegenerative tauopathies are being developed^26^. Consequently, characterisation of the effects of PERK downregulation in RPE cells in response to ER stress is required in order to provide insights for potential developments of similar future AMD treatment approaches.

In this context, we hypothesised that PERK downregulation dysregulates the UPR, apoptotic, antioxidant and autophagic responses in the RPE in response to ER stress^16,19^. Thus, the present study investigated the functional consequences of PERK downregulation on apoptosis, oxidative stress response and autophagy following brefeldin A (BFA)- and serum starvation-induced ER stress in the immortalised RPE cell line, ARPE-19. The findings indicate that under conditions of ER stress, PERK downregulation impairs the UPR, induction of apoptosis and the cells’ response to oxidative stress, with autophagic flux and autophagy induction being further impaired in response to serum starvation. Collectively, the data indicates that PERK downregulation impairs key RPE processes that contribute to AMD development.

## Results

### PERK downregulation increases ER stress through impaired UPR

**Figure 1:**
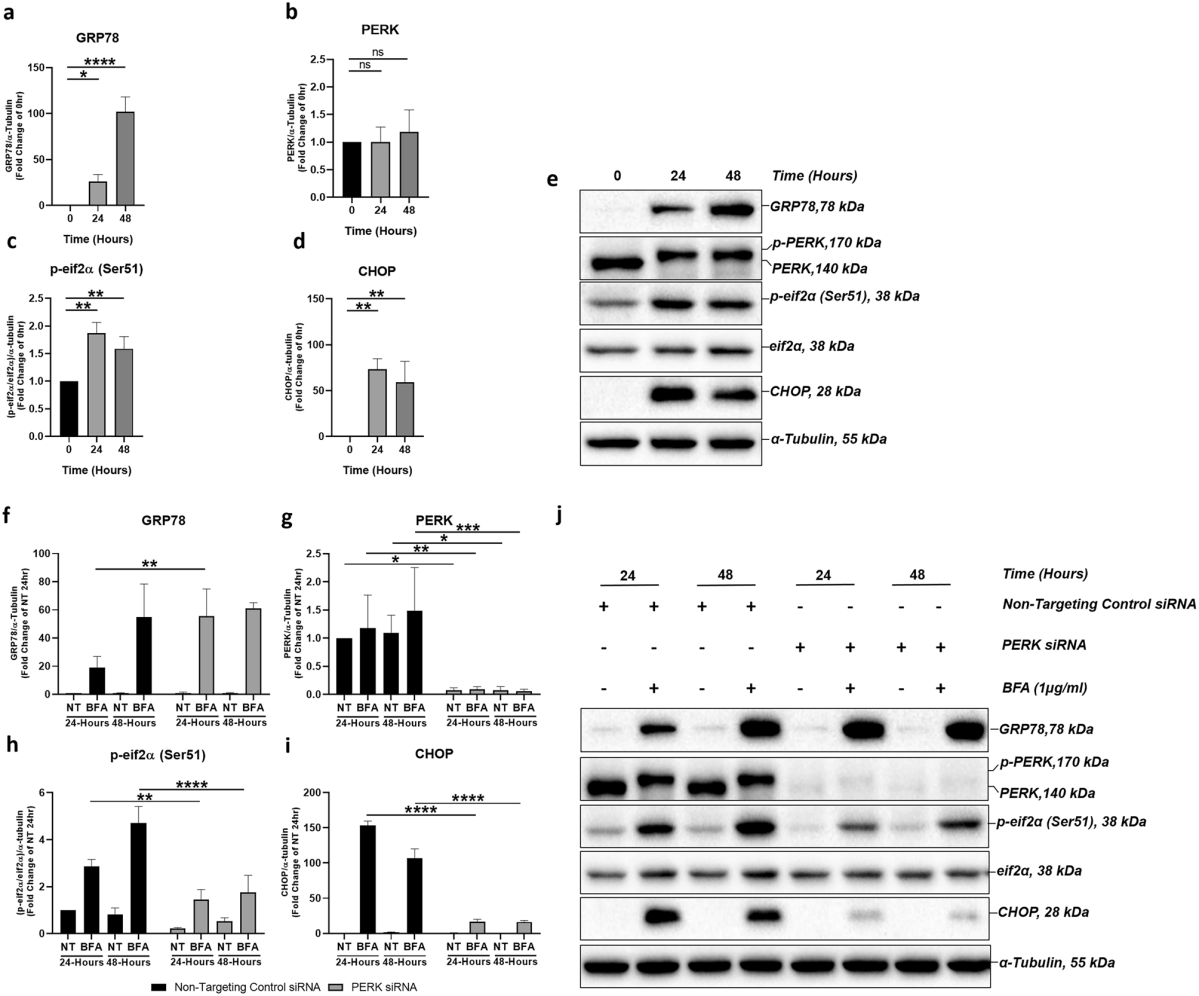
PERK downregulation increases ER stress. PERK signalling pathway mediates induction of ER stress in ARPE-19 cells treated with 1 μg/ml BFA through increased expression of GRP78 (**a**), PERK (**b**), p-eif2α (Ser51)/eif2α (**c**) and CHOP (**d**); representative immunoblots shown at 24-h and 48-h time-points (**e**). BFA-induced ER stress in cells in which PERK is downregulated with siRNA leads to significant upregulation of GRP78 (**f**) at 24-h time point and significant downregulation of PERK pathway components PERK (**g**), p-eif2α (Ser51)/eif2α (**h**) and CHOP (**i**) at 24 and 48-h time points when compared to non-targeting control siRNA; (**i**) representative immunoblots shown. Phosphorylation of PERK was observed from the slower migration of a total PERK band at 170 kDa following treatment with BFA (**b**,**g**,**e**,**j**). Phosphorylation of eif2α at serine 51 (p-eif2α (Ser 51)) was calculated relative to total eif2a protein expression (**c**,**h**). Results are presented as fold-changes of the 0-h time point (**a**–**d**) or the NT group in cells transfected with non-targeting control siRNA (**f**–**i**). Protein levels were normalised to α-tubulin. Means ± standard deviation are shown (n = 3). Statistical analysis was performed using a one-way ANOVA with Dunnet’s post-hoc test (**a**–**d**) or two-way ANOVA with Sidak’s multiple-comparison test (**f**–**i**) (*p ≤ 0.05, **p ≤ 0.01, ***p ≤ 0.001, ****p ≤ 0.0001). *BFA* Brefeldin A, *NT* non-treated, *NS* non-significant.

We first assessed whether ER stress and PERK pathway signalling can be induced in ARPE-19 cells by treating the cells with 1 µg/ml BFA for a time-course of 0–48 h (Fig. 1a–e). Induction of ER stress was confirmed by time-dependent upregulation of GRP78 from the 24-h time point, increasing further at 48-h (Fig. 1a,e). We next investigated whether BFA-induced ER stress initiated canonical PERK pathway signalling in ARPE-19 cells by assessing phosphorylation of PERK (Fig. 1b,e). Phosphorylation of PERK was assessed using the slower migration of total PERK detected by a band at 170 kDa as previously established^8^. Total PERK protein expression did not increase during BFA-induced ER stress, however we observed slower migration of the 170 kDa PERK band, suggestive of PERK phosphorylation at 24- and 48-h time points compared to non-treated (NT) samples at the 0-h time point (Fig. 1b,e). We then examined downstream effectors of PERK including phosphorylation of eif2α at serine 51 relative to total eif2α (p-eif2α (Ser51)/eif2α) (Fig. 1c,e) and CHOP (Fig. 1d,e) protein expression. Both displayed significant time-dependent increase at 24 and 48 h, compared to NT samples at the 0-h time point (Fig. 1c,d). Together this confirmed that ER stress can be induced via canonical PERK signalling pathway in ARPE-19 cells. Next, to assess the role of PERK in regulating the ARPE-19 ER stress-induced response, we carried out and confirmed siRNA-mediated PERK knockdown (Fig. 1g,j). siRNA-mediated PERK knockdown was carried out in the presence of BFA-induced ER stress for a time-course of 0–48 h. This was accompanied by a significant downregulation in downstream p-eif2α (Ser51)/eif2α (Fig. 1h,j) and CHOP expression (Fig. 1i,j) at 24- and 48-h time points. BFA-induced ER stress led to increased GRP78 protein expression following PERK knockdown at the 24-h time point, which was sustained at 48-h time point (Fig. 1f,j). Increased GRP78 expression following downregulated PERK pathway signalling suggests an increase in the requirement for ER chaperone-mediated degradation^8^, potentially as a result of increased ER-protein load due to a loss of global translation attenuation following decreased eif2α phosphorylation^8^. Together, these data indicate that ER stress is increased and the overall adaptive UPR is impaired following PERK downregulation in response to BFA-induced ER stress in ARPE-19 cells.

### PERK downregulation impairs ER stress-induced apoptosis in a Bax/Bcl-2-dependent manner

**Figure 2:**
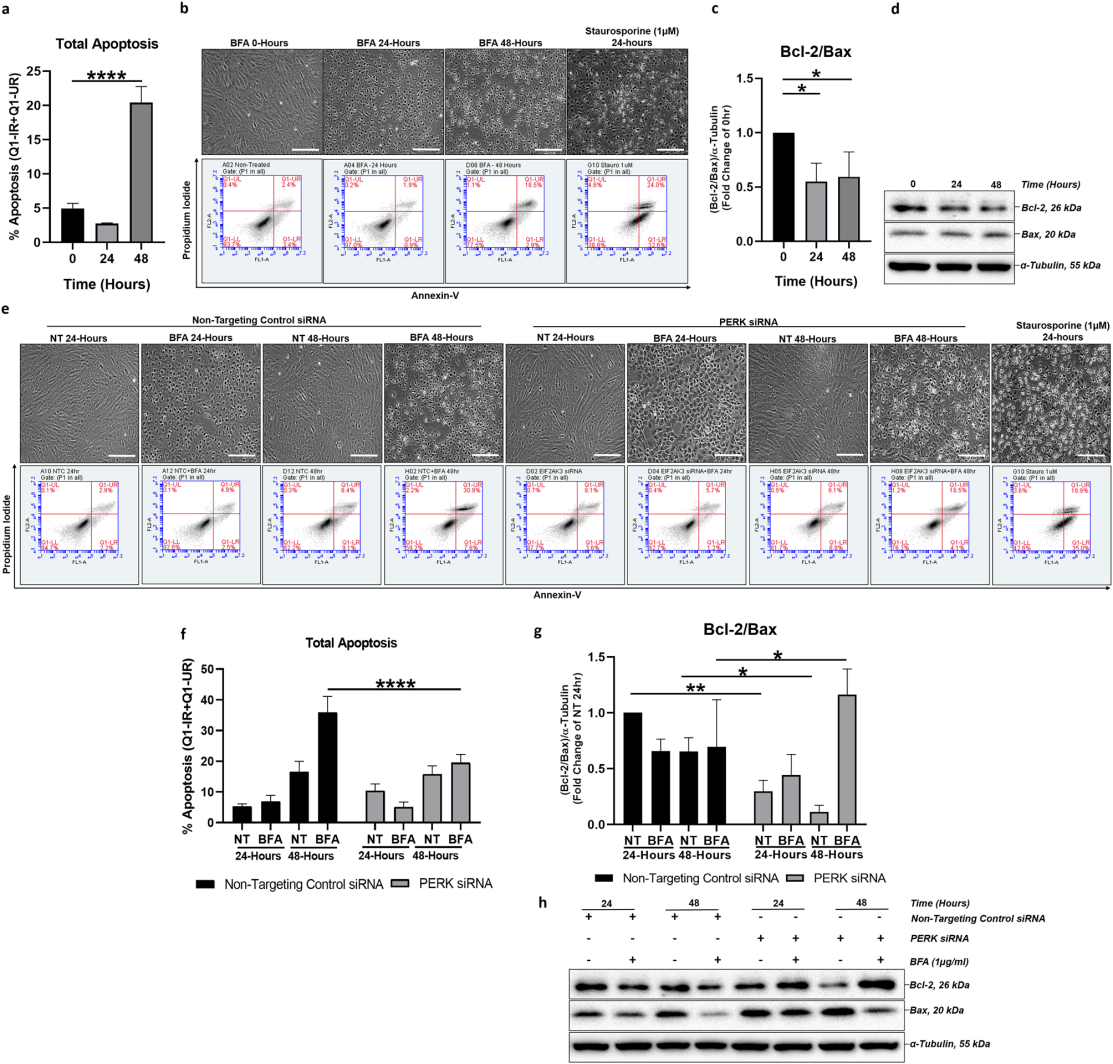
PERK downregulation impairs ER-stress induced apoptosis in a Bax/Bcl-2-dependent manner. Apoptosis associated with BFA-induced ER stress is mediated via PERK pathway signalling and involves downregulation of the Bcl-2/Bax ratio in ARPE-19 cells. Following PERK knockdown, ER stress-induced apoptosis is downregulated and associated with an increase in the Bcl-2/Bax ratio in ARPE-19 cells. Apoptosis of ARPE-19 cells treated with 1 μg/ml BFA for 0–48 h was analysed by flow cytometry using 1 μM Staurosporine as a positive control and Annexin V (AV) and Propidium Iodide (PI) as markers of early-stage and late-stage apoptosis, respectively. Total apoptosis was determined by combining the percentage of cells detected in the respective quadrants (lower right and upper right). Significantly increased ER stress-induced total apoptosis was determined by 48 h (**a**,**b**). Light microscopy showed ARPE-19 cells with apoptotic morphology by 24 h with large regions lacking adherent cells present by 48 h (**b**-top row). Under the same conditions, ARPE-19 cells showed significant decrease of the Bcl-2/Bax (anti-apoptotic/pro-apoptotic) proteins ratio (**c**); representative immunoblots shown (**d**). Following PERK knockdown, the percentage of cells undergoing ER stress-induced apoptosis decreased significantly compared to cells transfected with non-targeting control siRNA and treated with BFA by 48-h time point (**e**,**f**); representative light microscopy images (**e**, top row) and two parameter histogram plots with P1 gate applied (**e**, bottom row) shown. 24-h 1 µM Staurosporine was used as a positive control. Furthermore, in cells transfected with PERK siRNA, the ratio of Bcl-2/Bax protein expression showed a significant increase 48-h after induction of ER stress compared to cells transfected with non-targeting control siRNA, while significant decreases were determined in non-treated groups at both 24 and 48-h time points (**g**,**h**), with representative immunoblots shown (**h**). Results are presented as fold-changes from 0-h time-point (**a**,**c**) or fold changes from the NT group in cells transfected with Non-Targeting Control siRNA at a 24 h time-point (**f**,**g**). Representative light microscopy images, ×10 magnification, scale bar = 250 μm. Protein levels were normalised to α-tubulin. Means ± standard deviation are shown (n = 3)**.** Statistical analysis was performed using either the (**a**,**c**) one-way ANOVA with Dunnet’s post hoc-test or (**f**,**g**) two-way ANOVA with Sidak’s multiple-comparison test (*p ≤ 0.05, **p ≤ 0.01, ****p ≤ 0.0001). *NT* non-treated, *BFA* Brefeldin A.

In conditions of chronic ER stress, the pro-survival UPR switches to PERK-dependent apoptosis via upregulation of CHOP^19^. The initial experiments established that BFA initiates a time-dependent upregulation of pro-apoptotic CHOP expression (Fig. 1d,e), which is downregulated following PERK knockdown (Fig. 1i,j), confirming that CHOP is regulated in a PERK-dependent manner in ARPE-19 cells. Therefore, we hypothesized that PERK regulates ER stress-induced apoptosis in ARPE-19 cells. Significantly increased apoptosis was detected by flow cytometry following BFA-induced ER stress by 48-h time point (Fig. 2a,b), with cells starting to display specific apoptotic morphology by 24-h (Fig. 2b-top row). Using the Bcl-2/Bax ratio as an established measure to determine cellular sensitivity to apoptotic stimuli in RPE cell models^27^, we also observed that the Bcl-2/Bax ratio decreased in a time-dependent manner upon BFA-induced ER stress in ARPE-19, supporting the finding that the induced ER stress is associated with increased pro-apoptotic signalling (Fig. 2c,d).

We next investigated the role of PERK in regulating ER stress-induced apoptosis following BFA treatment. ARPE-19 cells were transfected with PERK siRNA or a non-targeting control siRNA followed by a 0–48-h time course of 1 μg/ml BFA. Following BFA treatment and PERK knockdown, ARPE-19 cells presented a more enlarged, cuboidal morphology with some rounding at 24 h when compared to the non-targeting control siRNA group (Fig. 2e-top row). Further, at 48 h the monolayer presented fewer regions devoid of cells in PERK knockdown ARPE-19 cells (Fig. 2e-top row). Flow cytometry revealed a significant decrease in total apoptosis in ARPE-19 cells transfected with PERK siRNA when treated with BFA compared to non-targeting control siRNA at a 48-h time-point (Fig. 2f). We further found that there was a significant increase in Bcl-2/Bax protein expression ratio in PERK knockdown cells at the 48-h time point (Fig. 2g,h). These data suggest that PERK regulates the rate of apoptosis over a 48-h time course by sensitising ARPE-19 cells to programmed cell death in a Bcl2/Bax-dependent manner.

### PERK downregulation impairs the ER stress-induced antioxidant response through downregulated Nrf2 signalling

The RPE is subjected to a high oxidative burden^28^. A central part of maintaining RPE redox homeostasis is upregulation of antioxidant Nrf2 signalling in response to oxidative stress^29^. Oxidative stress has been shown to upregulate ER stress signalling in RPE and PERK has been shown to directly phosphorylate Nrf2 in response to ER stress^9,10^. Therefore, we hypothesised that PERK may regulate the antioxidant response to BFA-induced ER stress in ARPE-19 cells.

**Figure 3:**
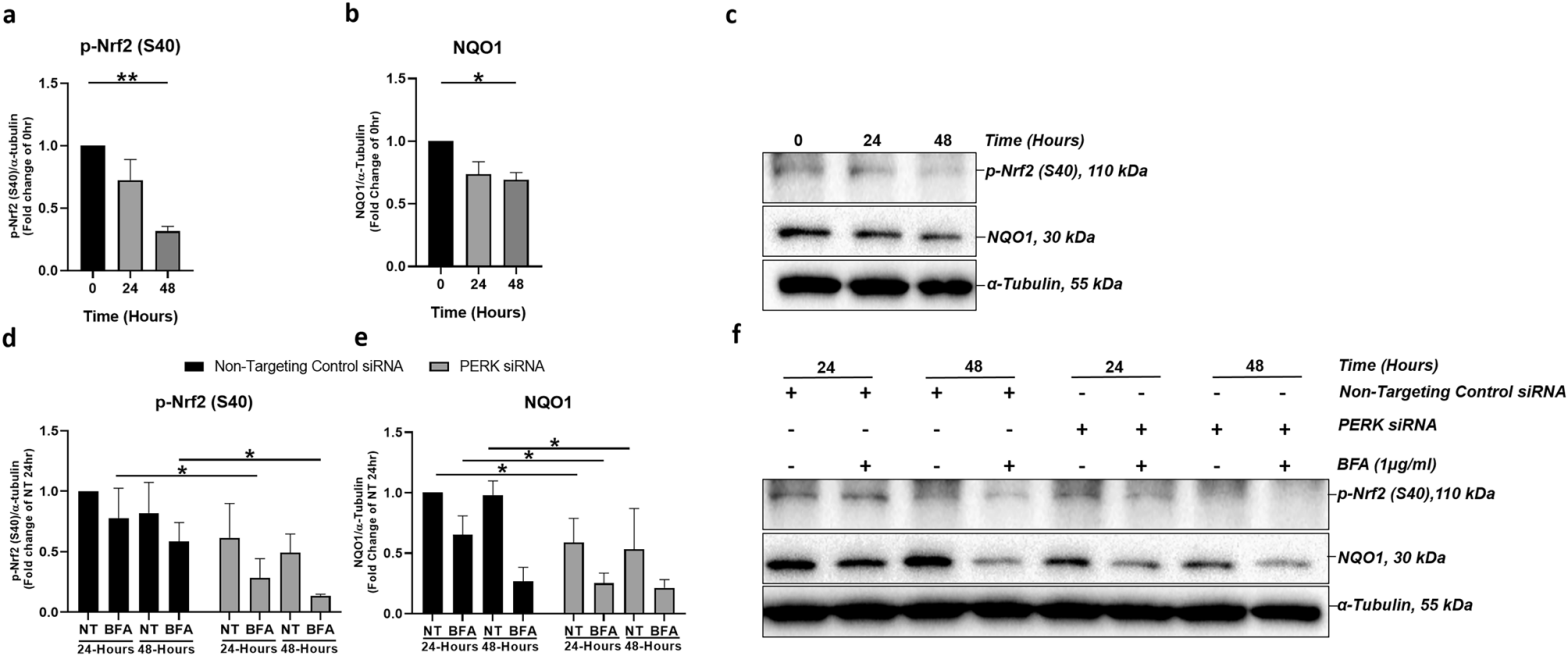
PERK downregulation impairs Nrf2 signalling and the response to BFA-induced ER-stress in ARPE-19 cells. ARPE-19 cells treated with 1 μg/ml BFA for 0–48 h resulted in a significant downregulation of (**a**) p-Nrf2 (S40) at 48 h and (**b**) NQO1 protein expression at 24 and 48-h time-points; (**c**) representative immunoblots shown. When ER stress was induced in APRE-19 cells in the presence of PERK siRNA there was a significant downregulation in (**d**) p-NRF2 (S40) at 24-h and 48-h in addition to (**e**) NQO1 at a 24-h time point compared to non-transfected control siRNA groups. When transfected with PERK siRNA, there was also a reduction in NQO1 protein expression in NT groups at 24 h and 48-h time points when compared to NT groups transfected with non-targeting control siRNA. (**f**) Representative immunoblots shown. Results are presented as fold-changes from 0-h time point (**a**–**c**) or the NT Non-Targeting Control siRNA group at a 24 h time point (**d**–**f**). Protein levels were normalised to α-tubulin. Means ± standard deviation shown (n = 3). Statistical analysis was performed using either the one-way ANOVA with Dunnet’s multiple-comparison test (**a**,**b**) or two-way ANOVA with Sidak’s multiple-comparison test (**d**–**e**) (*p ≤ 0.05, **p ≤ 0.01, ***p ≤ 0.001, ****p ≤ 0.0001). p-Nrf2 (S40) = Nrf2 phosphorylated at serine 40. *NT* non-treated, *BFA* Brefeldin A.

Firstly we investigated Nrf2 phosphorylation at serine 40 (p-Nrf2 (S40)) and NQO1 expression during a 48-h time-course of BFA treatment (Fig. 3a–c) and observed significant time-dependent decreases in p-Nrf2 (S40) (Fig. 3a,c) and NQO1 (Fig. 3b,c). The role of PERK in regulation of the antioxidant response mounted in the same conditions of ER-stress was investigated in PERK-knockdown ARPE-19 cells, which were unable to induce phosphorylated Nrf2 at 24-h and 48-h time points compared to ARPE-19 cells transfected with non-targeting control siRNA (Fig. 3d,f). Following PERK knockdown, NQO1 protein expression was significantly decreased even in the absence of additional ER stress and showed a further significant decrease following BFA-induced ER stress (Fig. 3e,f). Taken together, these data confirm the role of PERK in activation, through phosphorylation of Nrf2 and further demonstrate that downregulation of PERK dysregulates the response to oxidative stress in ARPE-19 cells mediated by the PERK-Nrf2 signalling axis.

### PERK modulates the rate of autophagy induction in ARPE-19 cells following ER stress

Autophagy is regulated by sustained ER-stress and PERK pathway signalling was previously shown to be implicated in this process P19 embryonic carcinoma cells constitutively expressing *c-jun* (C2C5 cells)^25,30^. Having confirmed a regulatory role for PERK in ER-/oxidative stress responses and related apoptosis modulation in ARPE-19 cells, we sought to investigate the role of PERK in regulation of autophagy following BFA-induced ER stress in the presence and absence of the lysosomal inhibitor chloroquine (CQ).

**Figure 4:**
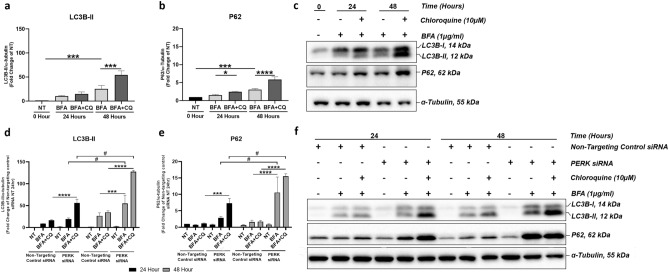
PERK regulates the induction of autophagy in ARPE-19 cells following ER stress. ER stress induced with 1 μg/ml BFA in ARPE-19 cells led to significantly increased protein levels of LC3B-II (**a**) at 48-h time point and P62 (**b**) at 24- and 48-h time points. Combined lysosomal inhibition using 10 μM CQ and 1 μg/ml BFA-induced ER-stress further increased LC3B-II and P62 protein expression at 24 and 48-h time points. (**c**) Representative immunoblots of BFA/CQ-treated ARPE-19 cells. When ER stress was induced in the presence of PERK siRNA, there was a significant increase in both LC3B-II (**d**) and P62 (**e**) at 48-h time point, with further significant increases in levels of both proteins in the presence of CQ when compared to non-targeting control siRNA groups. (**f**) Representative immunoblots of BFA/CQ-treated ARPE-19 cells, with and without PERK downregulation through siRNA knockdown. Results are presented as fold changes from the NT 0-h time point (**a**,**b**) and from the NT non-targeting control siRNA, 24-h time point (**d**,**e**). Protein levels were normalised to α-tubulin. Means ± standard deviation shown (n = 3). Statistical analysis was performed using a one-way ANOVA with Sidak’s multiple comparison test (**a**,**b**). Statistical analysis comparing treatment groups within each respective 24- and 48-h time point used a two-way ANOVA with the TukeyHSD post-hoc test (**d**,**e**) (*p ≤ 0.05, ***p ≤ 0.001, ****p ≤ 0.0001). Statistical analysis comparing groups across time-points used a two-way ANOVA with Sidak’s multiple comparison test (# = ****p ≤ 0.0001). *NT* Non-treated, *BFA* Brefeldin-A, *CQ* Chloroquine.

Upon induction of ER stress, LC3B-II and P62 (Fig. 4a–c) protein levels significantly increased in ARPE-19 cells at 48-h time point compared to the non-treated control. This indicated autophagosome accumulation resulting from impaired autophagic flux, either as a result of increased autophagy induction, or inhibition of lysosomal degradation^31^. However, concomitant use of BFA and CQ synergistically increased P62 protein expression at 24- and 48-h time points (Fig. 4b,c) and LC3B-II at 48-h time point (Fig. 4a,c), thus indicating that the induced ER stress and subsequent UPR upregulate autophagosome formation as opposed to inhibiting lysosomal degradation. The role of PERK in modulation of the autophagic flux was then investigated, under the same conditions, in PERK knockdown ARPE-19 cells. Thus downregulation of PERK led to significantly increased expression of both LC3B-II and P62 (Fig. 4d–f) at each time point when cells were incubated with both BFA and CQ, and when compared to non-targeting control siRNA groups treated with BFA only. In addition, when comparing 24-h and 48-h time points there was a significant time-dependent increase in LC3B-II and P62 (Fig. 4d,f) protein expression in cells concomitantly treated with BFA and CQ. These data suggest that PERK regulates the rate of autophagy induction in a time-dependent manner.

### PERK knockdown impairs the induction of autophagy in ER-stressed ARPE-19 cells in response to serum starvation

**Figure 5:**
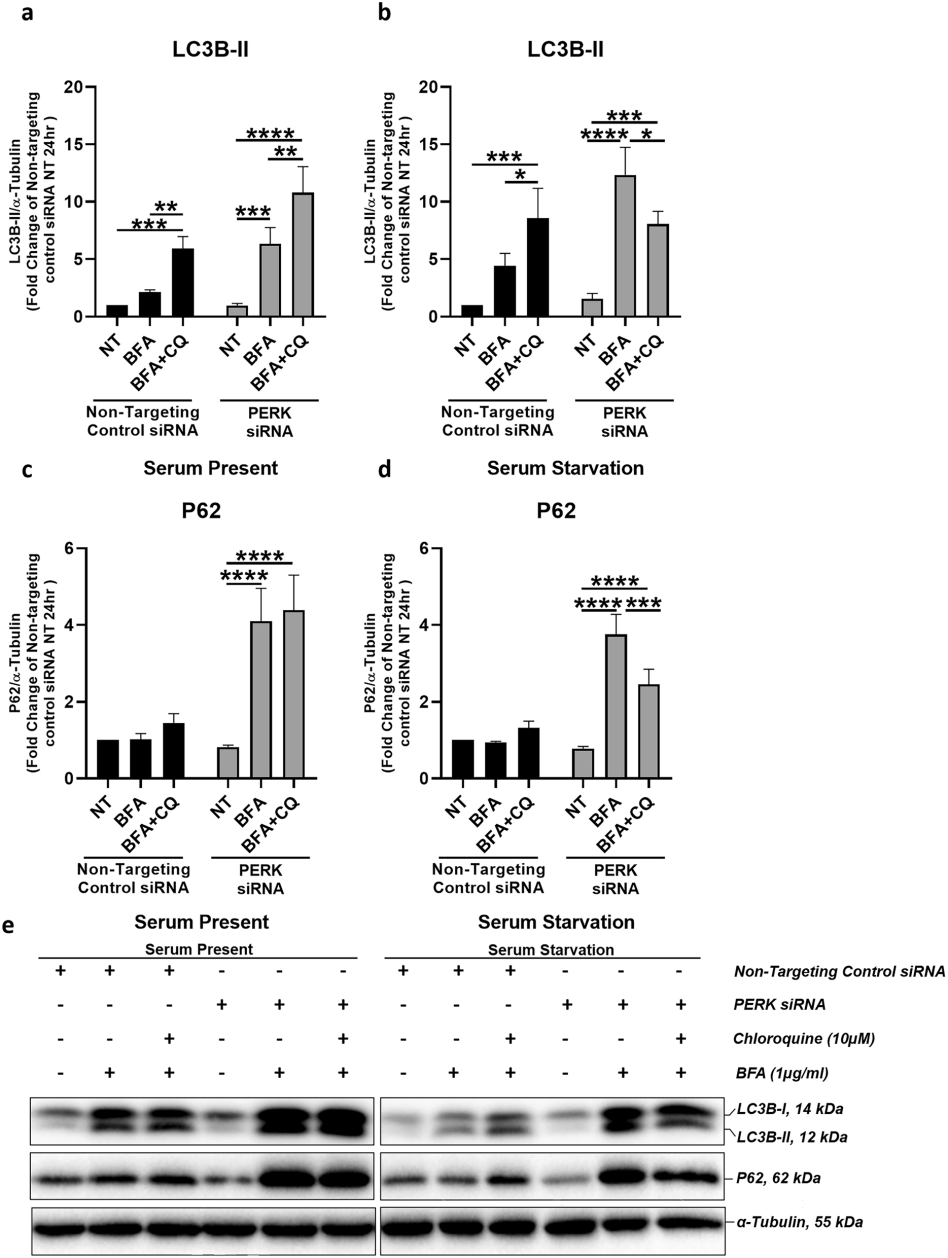
ER stressed ARPE-19 are unable to induce autophagy in response to serum starvation following PERK knockdown. ARPE-19 cells were subjected to 1 μg/ml BFA-induced ER stress with lysosome inhibition using 10 μM CQ for 24 h in the presence of PERK siRNA and non-targeting control siRNA to assess protein expression of LC3B-II (**a**,**b**) and P62 (**c**,**d**) in serum or serum starvation conditions, respectively. Consistent with previous results, there was a significant increase of LC3B-II protein level in BFA + CQ compared to both BFA and NT groups in cells transfected with non-targeting control siRNA, both in the presence and in the absence of serum. However, following PERK knockdown expression, while a significant increase in LC3B-II expression is observed in BFA + CQ compared to BFA groups in serum present conditions, there is a significant decrease in serum absent conditions. In (**c**) serum present conditions P62 displays a significant increase in expression with regards to the BFA + CQ and BFA alone groups compared to NT groups in cells transfected with PERK siRNA. No difference when comparing P62 expression between BFA + CQ and BFA alone. However, there was a significant decrease in P62 expression when comparing BFA + CQ and BFA groups in serum starvation conditions (**d**). Thus, in response to serum starvation, PERK knockdown ARPE-19 are unable to induce autophagy in conditions of ER stress. Protein levels were normalised to α-tubulin. Means ± standard deviations shown (n = 3). Statistical analysis was performed using a two-way ANOVA with TukeyHSD post-hoc test by comparing NT and BFA alone and combined BFA + CQ groups, within the non-targeting control siRNA and PERK siRNA group, respectively (*p ≤ 0.05, **p ≤ 0.01, ***p ≤ 0.001, ****p ≤ 0.0001). *NT* Non-treated, *BFA* Brefeldin-A, *CQ* Chloroquine.

It was previously demonstrated that in the pathological scenario of AMD, autophagy could not be induced by serum starvation in RPE cells^24^. Having established that PERK is essential for the rate of autophagy induction in response to ER stress and that PERK knockdown ARPE-19 cells display impaired autophagic flux, we assessed whether PERK knockdown ARPE-19 cells are capable of inducing autophagy when challenged with serum starvation and ER stress. We carried out PERK knockdown in ARPE-19 cells followed by ER stress induction using BFA under serum present (basal) or serum starved (autophagy induced) conditions. This was performed in the presence or absence of CQ pre-treatment and assessed at a 24-h time point (Fig. 5).When ARPE-19 cells were treated with BFA and CQ (BFA + CQ group) to concurrently induce ER stress and inhibit the lysosome under serum present conditions, there was a significant increase in LC3B-II (Fig. 5a,e) and a small but insignificant increase in P62 protein expression (Fig. 5c,e) compared to BFA without lysosome inhibition (BFA group) in PERK knockdown groups. Conversely, when autophagy was induced using serum starvation following PERK knockdown, a significant decrease in both LC3B-II (Fig. 5b,e) and P62 protein expression (Fig. 5d,e) was observed in samples when comparing cells concurrently treated with BFA and CQ (BFA + CQ group) to cells treated with BFA alone (BFA group). Therefore in a setting of serum starvation-induced autophagy, in addition to BFA-induced ER stress and CQ-induced lysosome inhibition, the significant reduction in LC3B-II and P62 expression observed suggests a relative reduction in autophagosome number, which likely reflects an inability of PERK knockdown ARPE-19 to induce autophagy.

## Discussion

This study aimed to investigate the molecular determinants that integrate ER stress-mediated pathways known to contribute to dysregulated cellular responses that lead to progressive RPE dysfunction in AMD. This is the first study to our knowledge that identifies an integrative role for PERK downregulation in increased ER stress, impaired autophagic flux, autophagy induction and Nrf2-mediated antioxidant response in a setting of ER stress, resulting from compromised proteostasis using BFA. Furthermore our results support previous findings of impaired ER stress-associated apoptosis^10^, which in the context of the data obtained in this study support a role for PERK downregulation in the impairment of RPE functions known to contribute to the development of AMD.

In this study we observed elevated GRP78 expression following PERK knockdown during BFA-induced ER stress. GRP78 acts as the initiating component of ER stress signalling and its upregulation functionally defines the UPR^23^. The UPR principally acts to attenuate global translation via PERK and eif2α phosphorylation^23^. Previous studies have determined that PERK knockdown in mouse embryonic fibroblasts (MEF) results in increased^35^ *S*-labeled cellular protein in the ER, indicative of an impaired attenuation of global translation and an increase of protein binding to GRP78 due to a loss of PERK kinase activity and eif2α phosphorylation^8^. In this context, our findings support the notion that upregulated GRP78 is an adaptive response intended on delaying induction of apoptotic pathways in ARPE-19 cells. Evidence for such a response was also obtained by studies demonstrating increased survival of chinese hamster ovary cells following GRP78 overexpression, supporting the pro-survival role of GRP78^32^. In the context of AMD, the RPE may display an impaired UPR in a setting of PERK downregulation, however further experimental evidence in human donor tissue or primary cell cultures is required.

Following PERK downregulation, ARPE-19 cells displayed impaired apoptosis induction associated with significant downregulation of CHOP expression. Our evidence of this protective effect corroborates previous findings which demonstrate that adenoviral-mediated PERK knockdown protects ARPE-19 cells from ER stress-associated apoptosis following hydroquinone and visible light exposure (400–800 nm)-induced ER stress via CHOP downregulation^10,11^. Indeed, CHOP has been shown to sensitise cells to apoptosis by downregulating gene expression of the anti-apoptotic protein Bcl-2 and upregulating pro-apoptotic Bax^22,33^. In human foetal RPE, tunicamycin-induced ER stress has been shown to downregulate the Bcl-2/Bax ratio and a further study in ARPE-19 cells has demonstrated regulation of the Bcl-2/Bax ratio in an x-box binding protein (XBP1)-dependent manner following cigarette smoke extract treatment^10,27^. Importantly, we identify that downregulation of PERK and subsequently CHOP regulates the Bcl-2/Bax ratio, which collectively suggests intrinsic apoptosis is induced via alterations in the Bcl-2/Bax ratio and that this is under the regulation of PERK in ARPE-19.

This study did not examine the well documented role of PERK activation resulting in cyclin D1 inhibition via phosphorylation of eif2α leading to cell cycle arrest^34^. Specifically, Brewer and Diehl posited that cell-cycle exit is part of the process of coordinating apoptosis initiation in ER stress and that PERK expression is key to the regulation of this process^34^. In this respect, while PERK knockdown was shown to increase sensitivity to ER-stress apoptosis in embryonic stem cells^8^, work from Chen et al. concluded that PERK knockdown in ARPE-19 cells results in apoptosis inhibition during ER stress^10^. This suggests that the role of PERK in regulating decisions of cellular fate is cell-type dependent. The work of Brewer and Diehl bought forth the theory that the induction of cell-cycle arrest by PERK after activation of the UPR may provide time necessary to re-establish cellular homeostasis. However, it is unclear as to whether this is true for the RPE and further experimentation is required to confirm the dynamics between PERK downregulation, cell-cycle arrest and apoptosis.

As downregulated *EIF2AK3* was documented in the RPE of AMD patients^16^, the findings outlined in this study in the model ARPE-19 system could suggest that in vivo, RPE in AMD may lose the ability to initiate the apoptotic program in response to accumulated misfolded proteins in the ER, possibly as a protective measure to ensure cellular survival of the post-mitotic RPE. However, this must be interpreted with caution as unlike the RPE in vivo, cultured ARPE-19 cells can be highly proliferative and are accompanied by a phenotype associated with de-differentiation^35^. This may explain why our findings using the immortalised ARPE-19 cell line differ from those reported by Dunaief et al., who described upregulated apoptosis from AMD RPE in vivo^36^. Therefore, while we suggest that the upregulation of apoptosis in AMD RPE is not directly or solely mediated by ER stress signalling, further experiments in primary donor tissue are required to confirm this effect. The RPE is subject to oxidative damage from both endogenous and exogenous pro-oxidant sources^28^. Nrf2 is a principal regulator of phase II antioxidant enzymes and detoxifying gene expression and displays decreased gene expression in AMD RPE^28,37^. PERK has been established as a direct kinase of Nrf2, facilitating Nrf2 phosphorylation and phase-II antioxidant NQO1 expression in response to ER stress^9,20^. As the UPR via PERK pathway signalling has been shown to increase oxidative stress, we reasoned that ARPE-19 cells are unable to induce an antioxidant response via Nrf2 signalling following PERK downregulation in a setting of BFA-induced ER stress^38^. However, in response to BFA-induced ER stress, we observed a time-dependent decrease in Nrf2 phosphorylation and NQO1 protein expression. Studies in RPE and MEF cells have shown an inverse regulatory relationship between Nrf2 and CHOP protein expression^20,39^. Hence it is possible that BFA-induced and time-dependent CHOP upregulation in this study may explain the decrease in Nrf2 signalling and resultant decrease in NQO1 expression at the same time-points. While previous studies in ARPE-19 cells have demonstrated that the Nrf2 signalling axis is regulated in an XBP1 dependent manner, we sought to establish whether this was true for PERK^40^. Importantly, in PERK knockdown ARPE-19 cells, we observed a decrease in both phosphorylated Nrf2 and NQO1 expression in the absence of ER stress, which was further reduced following ER-stress induction using BFA. Our data demonstrates that loss of PERK impairs the Nrf2-dependent antioxidant response to ER stress in ARPE-19 cells. While our findings are in line with previous evidence of PERK-dependent Nrf2 phosphorylation in MEF cells^9^, this is the first time to our knowledge that the PERK-dependent regulation of Nrf2 phosphorylation was demonstrated experimentally in ARPE-19 cells. In the context of previously observed *EIF2AK3* downregulation and loss of Nrf2 signalling in AMD RPE, it is possible that PERK downregulation could impair Nrf2 signalling in AMD RPE, leaving cells unable to maintain redox homeostasis in a setting of impaired global translation attenuation and impaired apoptosis in response to ER stress^16,37^.

PERK and its downstream signalling effectors have been shown to be essential in regulating the induction of autophagy in response to ER stress^41^. In ARPE-19 cells, autophagy can be induced by photooxidative or high-glucose environments that result in ER stress^11,42^. The impairment of autophagy has been extensively implicated in the ageing RPE and in the pathogenesis of AMD^24,43,44^. Our data show that combined BFA-induced ER stress and lysosomal inhibition using CQ result in a synergistic increase in LC3B-II and P62 expression compared to BFA treatment alone. This suggests that ER stress and induction of the UPR regulates the rate of autophagic induction and results in autophagosome formation exceeding that of lysosomal degradation, thus impairing autophagic flux in ARPE-19 cells^31^. Furthermore, we observed that PERK knockdown ARPE-19 cells displayed even greater increases in LC3B-II and P62 expression in the presence of BFA alone and BFA + CQ treatment compared to non-targeting control siRNA cells. This indicates a key role for PERK in the regulating autophagy induction. Early studies established that classical ER stress inducing agents such as thapsigargin and tunicamycin upregulate autophagosome accumulation in an ER stress-specific manner, playing a protective role in settings of ER stress^25,45–47^. As such, autophagy may represent a para-adaptive mechanism supportive of the UPR^25,45–47^. It has been previously established in MEF cells that during ER stress, protein influx into the ER continues following PERK knockdown^8^, which could explain why autophagy induction is dramatically increased and autophagy flux is impaired in PERK knockdown ARPE-19 cells. Thus, the requirement for autophagy as a supportive mechanism of the UPR becomes apparent, aiding in the clearance of a large amounts of accumulated protein from the ER^25,45–47^. Therefore, we suggest that BFA-induced ER stress regulates the induction of autophagy and that PERK regulates the rate of autophagy induction in ARPE-19 cells.

A recent study demonstrated that following nutrient starvation, primary RPE cells cultured from AMD donors were unable to induce autophagy^24^. As PERK pathway signalling was shown to be essential in regulating the induction of autophagy in MEF cells^41^, we sought to investigate whether PERK knockdown ARPE-19 cells were able to induce autophagy when challenged with serum starvation in a setting of BFA-induced ER stress. Under these conditions, we observed an increase in autophagosome formation, indicated by LC3B-II and P62 protein expression; however, following BFA-induced ER stress in the presence of CQ-induced lysosomal inhibition, both LC3B-II and P62 protein expression decreased compared to BFA treatment alone. If PERK knockdown ARPE-19 cells were competent in autophagy induction, a synergistic effect on the increase of LC3B-II would be expected following lysosomal inhibition^48,49^. Our findings show that when lysosomal degradation is inhibited using CQ, autophagosome formation is occurring at a lower rate, suggesting that PERK knockdown ARPE-19 cells are unable to induce autophagy in response to nutrient starvation. Furthermore, while an increase in P62 expression may indicate autophagosome accumulation and impairment^49^, a decrease could possibly reflect lower number of total autophagosomes formed as a result of impaired autophagy induction. These findings are in line with the observations of Mitter et al. who described significant reduction of LC3 in RPE in early and late-stage AMD compared to age-matched controls^43^.

A previous study examining the regulation of gene expression by DNA methylation in early-stage human donor AMD RPE revealed that *EIF2AK3* (encoding PERK) gene expression is downregulated, although not associated with differential DNA methylation^16^. Unanswered questions remain as to whether *EIF2AK3* downregulation acts as an adaptive mechanism to ageing-related alterations which ultimately result in RPE dysfunction in late-stage disease. Indeed, inhibited apoptosis following PERK downregulation in a setting of BFA-induced ER stress observed in this study does not reflect upregulated apoptosis observed in later stages of disease^36^. Therefore, it is possible that PERK is downregulated in earlier stages of disease as an adaptive mechanism to prevent cell death in the post-mitotic RPE.

The UPR is comprised of a highly complex and interconnected network of signalling cascades that are yet to be fully delineated^23^. In this context, the next steps for fully characterising the regulatory role of PERK downregulation in ARPE-19 and further in AMD RPE need to focus on examining the induction and downstream signalling pathways of the other two major components of UPR, the IRE1 and ATF6, that also regulate ER stress, apoptosis and autophagy following PERK knockdown^8,10,40^.

## Conclusion

The findings of this study evidence a key role for PERK and components of the PERK signalling pathway in maintaining RPE homeostasis. Our results in the context of previous studies suggest that the PERK-encoding *EIF2AK3* gene may be downregulated in early/intermediate AMD^16^ to protect cells from apoptosis as a result of mounting ER stress early in the disease course. We propose that following PERK downregulation, the compensatory UPR and cellular antioxidant defence system is impaired. However upregulated ER chaperone expression alone is unable to maintain proteostasis, therefore an inhibition of apoptotic signalling and significant increase in the rate of protective mechanisms such as autophagy are required to support misfolded protein clearance. However, it is possible that alone these compensatory mechanisms are unable to mitigate mounting cellular dysfunction leading to RPE apoptosis observed in late-stage AMD^36^. With clear evidence of its role in dysregulated ER stress signalling pathways in RPE cells, the PERK signalling pathway emerges as a critical component of the complex regulatory mechanisms influencing AMD pathogenesis and as such may provide avenues for future therapeutic interventions.

## Methods

### Cell culture

ARPE-19 cells are immortalised RPE cells established from the ocular globes of a 19-year old male donor as previously described^50^, were obtained from ATCC, USA. Cells were grown in a 1:1 mixture of Dulbeco Modified Eagles Medium and Ham’s F12 nutrient mixture (Sigma Aldrich, Dorset, UK) supplemented with 10% heat inactivated foetal bovine serum (FBS) (Sigma Aldrich, Dorset, UK), at 37˚C and 5% CO_2_ and passaged using 1× Trypsin–EDTA (Sigma Aldrich, Dorset, UK) at a 1:3 ratio. Cells were counted using cell dual-chamber cell counting slides (BioRad, USA) on a Bio-Rad TC10 automated cell counter (BioRad, USA), and seeded into 6-well cell culture plates (6-well plate) (Corning Incorporated, Maine, USA) at a seeding density of 2.5 × 10^5^ cells per well.

### Induction of endoplasmic reticulum stress

ARPE-19 were seeded in 6-well plates and incubated (37 °C, 5% CO_2_) for 48 h. ER stress induction was performed using an optimised concentration of 1 μg/ml (BFA) (Sigma Aldrich, Dorset, UK) for all experiments. BFA is a fungal metabolite that inhibits ADP ribosylation factor, resulting in reduction of coatomer protein assembly and inhibiting anterograde vesicular transport between the ER and Golgi apparatus resulting in protein accumulation in the ER lumen resulting in ER stress^51^. Cell lysates for protein expression analysis and treated ARPE-19 cells used in flow cytometry were collected at time points from 0 to 48 h post-treatment. For experiments involving siRNA transfection, ER-stress was induced following a 48 h incubation of ARPE-19 cells with transfection complexes.

### siRNA transfection

The *EIF2AK3* siRNA-duplex (s18102, Invitrogen, USA) (5 nM) was delivered using 0.1% w/v Lipofectamine™ RNAiMAX Transfection Reagent (ThermoFisher Scientific, USA) in Opti-MEM™ Reduced Serum Medium (ThermoFisher Scientific, USA). All transfections included a non-targeting control siRNA using 10 nM Silencer™ Select Negative Control No. 1 siRNA (Invitrogen, USA) and *EIF2AK3* transfection complexes were reverse transfected into ARPE-19 cells as per the manufacturer’s instructions. The ARPE-19 cells were incubated with transfection complexes for 48 h at 37 °C. Following incubation, cell lysates were collected for analysis or subjected to the desired treatment, after which cell lysates were collected for analysis of protein expression using western immunoblotting or flow cytometry.

### Autophagy flux assay

Lysosome inhibition was carried out using CQ (10 µM) (Sigma Aldrich, Dorset, UK) in complete media or serum free Dulbeco Modified Eagles Medium and Ham’s F12 nutrient mixture. ARPE-19 cells were pre-treated with 10 µM CQ for 24 h to ensure lysosome inhibition, before removing the media and washing each well twice with 37 °C distilled phosphate buffered saline (1X) (dPBS) before BFA-induced ER stress. For experiments involving assessing autophagy flux following siRNA transfection, ARPE-19 cells were incubated with transfection complexes for 48-h before a 24 h pre-treatment with 10 μM CQ. Thereafter, ER stress was induced using BFA for 0–48 h as previously stated. For experiments involving autophagy induction using serum starvation and siRNA transfection, ARPE-19 cells were seeded in 6-well plates and grown for 48 h in complete media and lysosome inhibition carried out as previously stated. Each well was then washed twice with dPBS (37 °C) to remove remaining serum, followed by treatments involving ER stress induction in serum-free DMEM F12 and complete media for time-courses of 0–48 h. Cell lysates were then collected for protein expression analysis using western immunoblotting.

### Apoptosis induction

Apoptosis was induced in ARPE-19 cells by inducing ER stress using 1 μg/ml BFA for a time-course of 0–48 h. Cell lysates were collected for protein expression analysis using western immunoblotting or prepared for further analyses using flow cytometry from 0 to 48 h post-treatment. ARPE-19 were treated for 24-h with staurosporine (1 μM) (Sigma Aldrich, Dorset, UK) as a positive control for apoptosis in flow cytometry experiments.

### Flow cytometry

Following apoptosis induction, floating cells were collected, and the remaining adherent cells washed once in dPBS (37 °C) collecting the dPBS after to ensure all cells undergoing apoptosis were collected. Adherent ARPE-19 cells were detached using 1 × Trypsin–EDTA and collected. Phosphatidylserine (PS) externalisation was used to detect apoptosis induction using the Annexin V-FITC Apoptosis Staining/Detection Kit (Abcam, Cambridge, UK) by flow cytometry using 5 µl Annexin V (AV) (FITC) and 5 µl Propidium Iodide (PI) as per the manufacturer’s instructions. Stained experimental and unstained control cells were analysed using a BD Accuri C6 flow cytometer using four fluorescence detectors for each time point over a 0–48-h time-course. 50,000 events were collected per sample. Flow cytometry analysis was carried out using the BD Accuri C6 software with AV (FITC) analysed in Filter 1 (FL1) and PI (PE) analysed in Filter 2 (FL2). To account for spectral overlap when using AV and PI stains, colour compensation was applied. For experiments analysing apoptosis, the polygonal gating tool was used to select the population of intact (healthy) cells and non-intact (apoptotic) cells in the P1 Gate. To analyse the cell number positively stained with AV in non-intact cells, cells were separated into quadrants on a cytogram. The left lower quadrant indicates viable cells. The right lower quadrant represents AV positive/PI negative staining containing cells undergoing early apoptosis only. The right upper quadrant indicates both high AV and PI staining, showing cells undergoing late-stage apoptosis. The left upper quadrant represents low AV and high PI staining, showing cells undergoing necrosis only. The combined percentage of cells present in the lower right (Q1-LR) and upper right (Q1-UR) quadrants in P1 gates was used to determine the percentage of total apoptosis in the population of ARPE-19 cells tested.

### SDS-PAGE and western immunoblotting

ARPE-19 cell lysates were prepared for western immunoblotting by washing wells in a 6-well plate twice with dPBS and scraping wells in lysis buffer (0.128 M β-mercaptoethanol, 40 mM tris, 10% glycerol, 1% SDS, 0.01% bromophenol blue). The cell lysate was homogenised on ice carefully using a 21G needle and incubated (95 °C, 5 min) to ensure sample denaturation. Western immunoblotting was performed as described previously^52,53^. Primary and secondary antibodies used in western immunoblotting are listed in Tables 1 and 2, respectively. Protein expression for experimental proteins of interest were normalised for loading using the loading control α-tubulin. For immunoblotting involving probing for P62 (SQSTM1) protein expression, membranes were stripped by incubating with stripping buffer (3 M Tris, 10% SDS, 0.128 M β-mercaptoethanol pH 6.7) (50 °C, 30 min) with gentle agitation and subsequently washed three times for 5 min in washing buffer (0.02 M Tris, 0.14 M NaCl, 0.1% Tween-20, pH 7.6) before being blocked in blocking buffer (0.02 M Tris, 0.14 M NaCl, 0.1% Tween-20, 5% (w/v) non-fat dry milk) and incubated in the respective primary antibody overnight for visualisation.

**Table 1:** Primary antibodies used for western immunoblotting.

Antibody	Supplier (code)	Description
PERK	Abcam (ab65142)	Rabbit polyclonal
eif2α	Cell signalling technology (#9722)	Rabbit polyclonal
eif2α pSer51	Cell signalling technology (#9721)	Rabbit polyclonal
GRP78 (BiP)	Cell signalling technology (#3177)	Rabbit monoclonal
Nrf2 pSer40	Abcam (ab76026)	Rabbit monoclonal
NQO1	Cell signalling technology (#3187)	Mouse monoclonal
SQSTM1/p62	Cell signalling technology (#5114)	Rabbit polyclonal
LC3B	Cell signalling technology (#2775)	Rabbit polyclonal
CHOP	Cell signalling technology (#2895)	Mouse monoclonal
Bax (D2E11)	Cell signalling technology (#5023)	Rabbit monoclonal
Bcl2	Abcam (ab32124)	Rabbit monoclonal
Alpha Tubulin	Abcam (ab4074)	Rabbit polyclonal

**Table 2:** Secondary antibodies used for western immunoblotting.

Antibody	Supplier (code)	Description	Dilution, Buffer
Anti-rabbit IgG	Sigma Aldrich (A0545)	Goat anti-rabbit IgG, HRP-linked	1:10,000, 5% Milk/TBST
Anti-mouse IgG	Sigma Aldrich (A9044)	Rabbit anti-mouse IgG, HRP-linked	1:10,000, 5% Milk/TBST

### Statistical analysis

Data generated in this study was analysed to determine statistical significance using the GraphPad Prism 8 (GraphPad Software, USA). Sample means were compared to control groups using One-way Analysis of Variance (ANOVA) with Dunnett’s or Sidak’s post hoc-test. When two or more treatments were measured alone or over a time-course, comparisons were made using a Two-way ANOVA with Sidak’s or TukeyHSD post-hoc test. All experiments consisted of n = 3 replicates, unless otherwise stated. All data values are presented as mean ± standard deviation and, when appropriate, as fold change of control groups for each experiment. *p*-values are shown in figure legends where *p ≤ 0.05, **p ≤ 0.01, ***p ≤ 0.001, ****p ≤ 0.0001.

## Supplementary Information

Supplementary Figures.

## Data Availability

All data generated or analysed during this study are included in this published article. Uncropped western immunoblot images used in the creation of Figs. 1, 2, 3, 4 and 5 in this study are provided in Supplementary Figs. 1–5 in Supplementary File 1. Original images and data generated for all individual replicates for each experiment during the current study are available from the corresponding author on reasonable request.
