# Supplementary figures and images for: PERK/EIF2AK3 integrates endoplasmic reticulum stress-induced apoptosis, oxidative stress and autophagy responses in immortalised retinal pigment epithelial cells
Source: Sci Rep. 2022 Aug 3;12:13324. doi: 10.1038/s41598-022-16909-6 (PMC9349321; doi:10.1038/s41598-022-16909-6)

## Slide 1
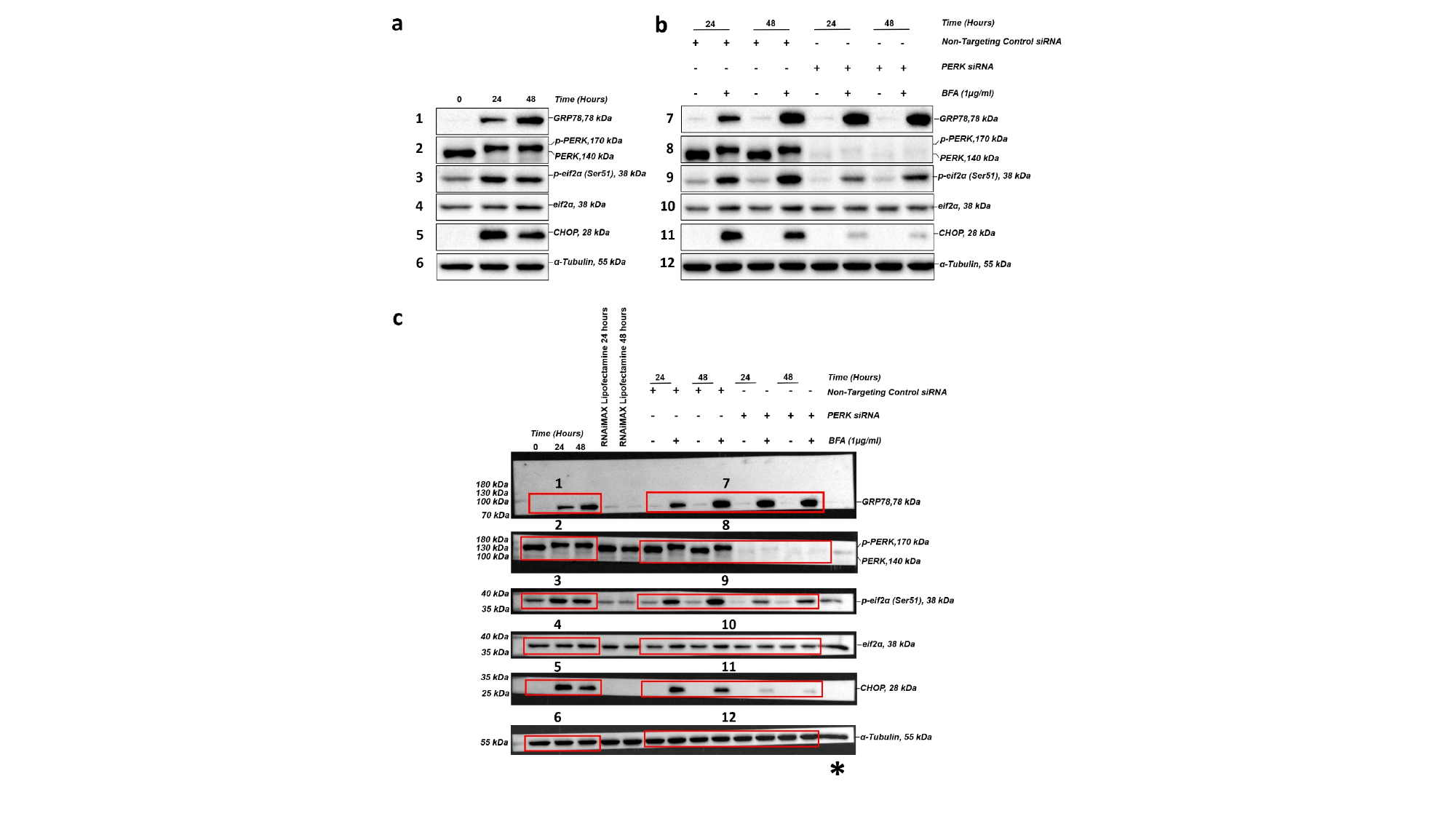

## Slide 2
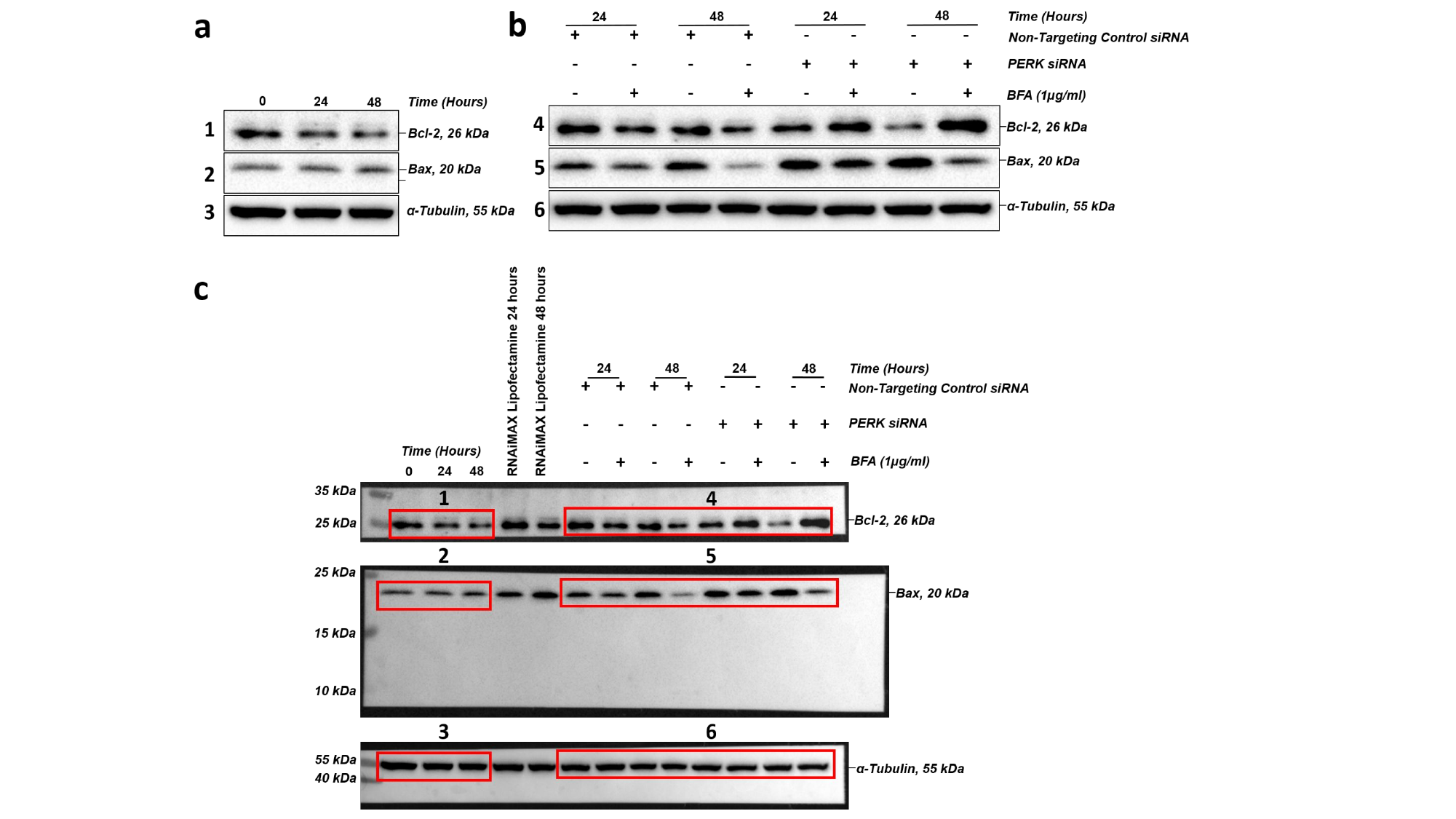

## Slide 3
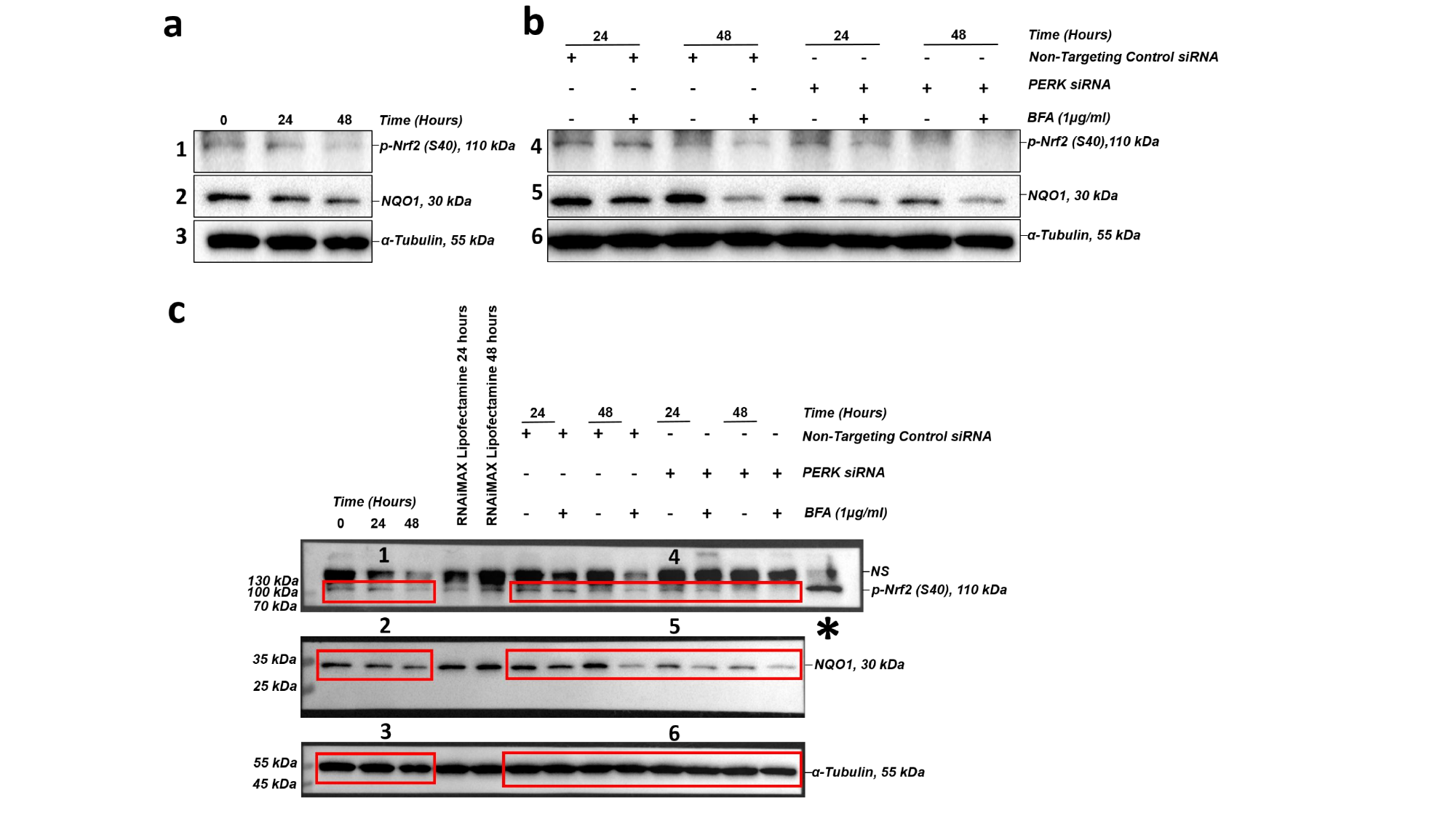

## Slide 4
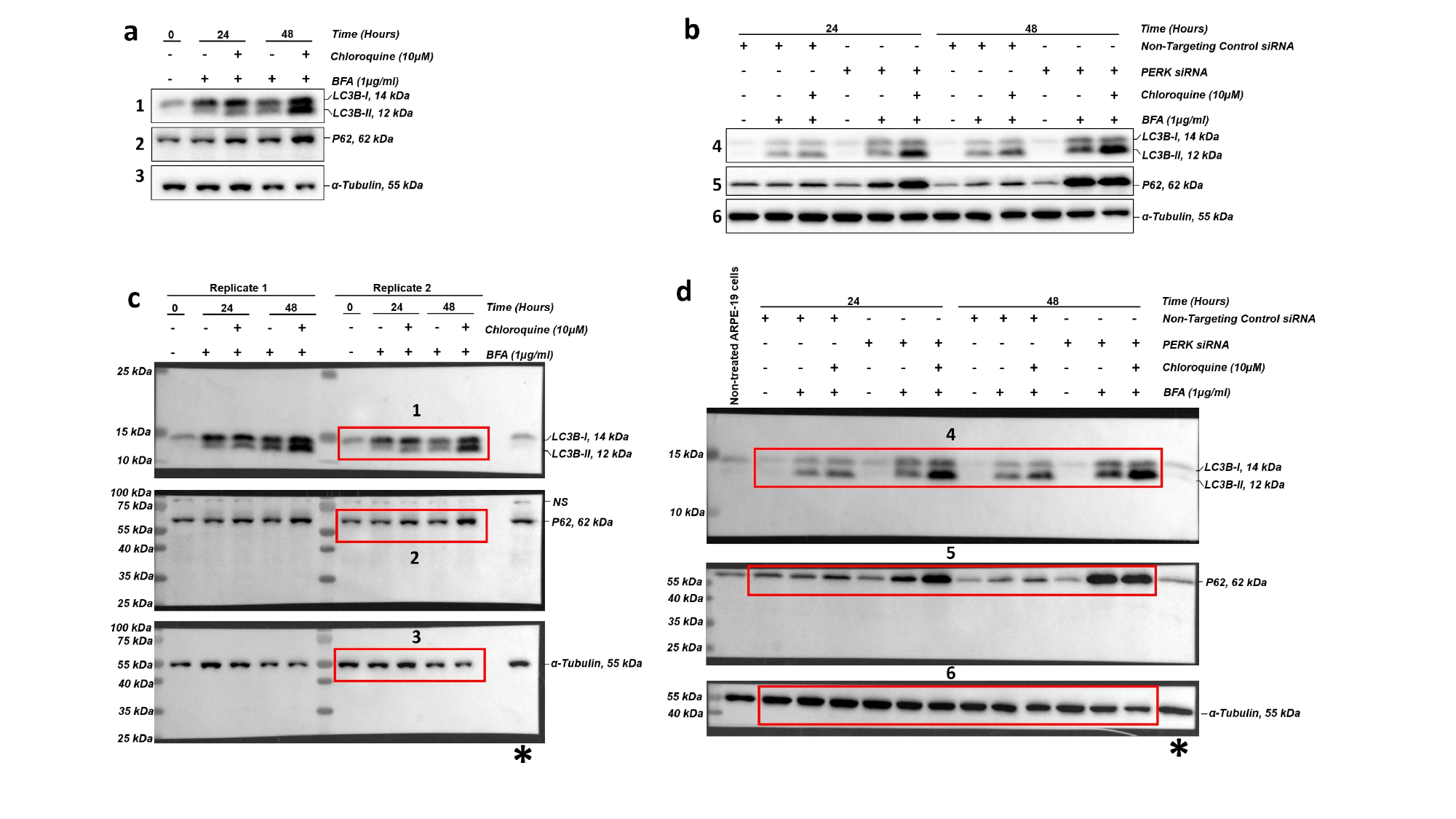

## Slide 5
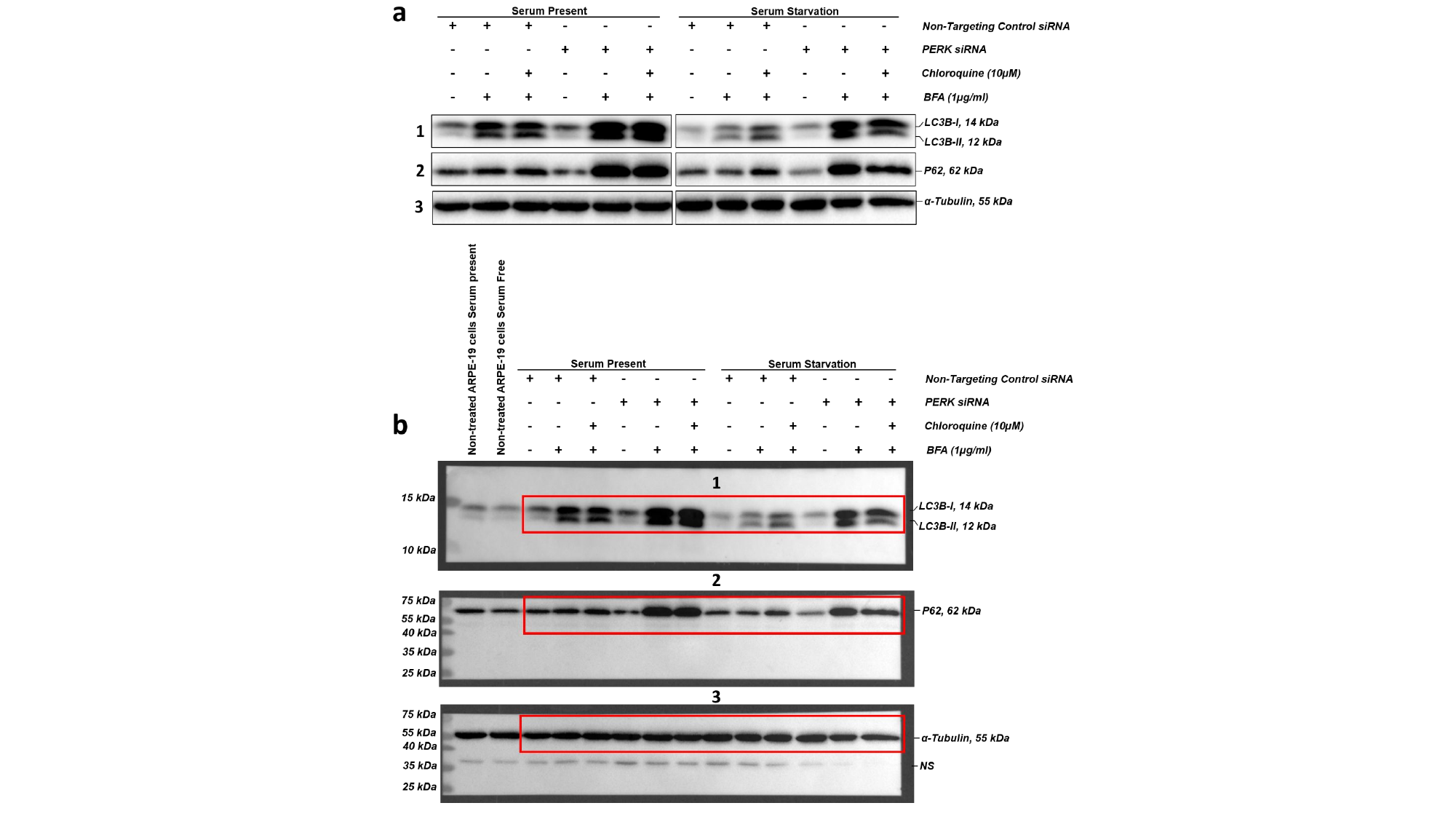

Supplement: Supplementary file 1 — Supplementary Figures. [file 41598_2022_16909_MOESM1_ESM.pptx]
